# Evaluating primary tumor position variation for rectal cancer patients treated with long course radiotherapy

**DOI:** 10.1088/1361-6560/adcaf8

**Published:** 2025-04-24

**Authors:** Dennis Tideman Arp, Ane L Appelt, Rasa Mikalone, Martin Skovmos Nielsen, Laurids Østergaard Poulsen

**Affiliations:** 1 Department of Medical Physics, Oncology, Aalborg University Hospital, Aalborg 9000, Denmark; 2 Department of Clinical Medicine, Aalborg University, Aalborg 9000, Denmark; 3 Leeds Institute of Medical Research at St James’s, University of Leeds, Leeds LS2 9JT, United Kingdom; 4 Leeds Cancer Centre, St James’s University Hospital, Leeds LS9 7TF, United Kingdom; 5 Department of Radiology, Aalborg University Hospital, Aalborg 9000, Denmark; 6 Department of Oncology, Clinical Cancer Research Center, Aalborg University Hospital, Aalborg 9000, Denmark

**Keywords:** rectal cancer, radiotherapy, systematic and random errors, positional variation, primary tumor, interfraction

## Abstract

*Objective.* To quantify interfraction shape and positional variations of primary tumor volumes for rectal cancer patients receiving long course radiotherapy by comparing two quantification strategies: a center-of-mass (COM) method and a surface-based metric that captures local deformations. *Approach.* This study utilized repeat MRI scans before and during radiotherapy (RT) for rectal cancer to investigate the positional variation of the primary gross tumor volume (GTVp). Sixteen patients underwent six MRI exams, with the initial three before the RT course and the subsequent three at one, two, and four weeks into the RT course. GTVp’s were delineated on 3D T2-weighted MRIs, and positional variation analyzed using both COM and point-based surface displacements against the initial scan. Surface displacements were quantified using a bidirectional local distance measure, analyzing 3D displacement vectors. Additionally, the study examined local right–left (RL) and anterior–posterior (AP) surface variations relative to tumor height in the rectum by mapping baseline GTVp volumes onto a reference rectum structure. *Main results.* Systematic error for COM measurements were 1.7, 1.3 and 2.0 mm for AP, RL, and cranial–caudal (CC) direction, respectively. Random errors were 2.1, 1.2 and 2.2 mm, while the GM errors were −0.3, 0.5 and −0.3 mm for AP, RL, and CC directions, respectively. An increase in systematic and random errors were observed when comparing 95th percentile surface displacements to the COM measurements, indicating local displacements which the COM did not detect. Additionally, a general tendency for higher-located tumors to experience larger left–right and AP surface variations were seen when evaluating the 95th percentile. *Significance.* COM-based analysis might underestimate local deformations. Consequently, surface-based methods might provide more robust estimations of systematic, random and group mean errors for planning target volume-margin calculation. The surface variations tend to increase for tumors located in the upper part of the rectum.

## 1. Introduction

Estimating the positional day-to-day variation of the treatment target is an integral part of standard radiotherapy (RT) planning and delivery, particularly for irregular and deformable tumors which exhibit complex surface movement patterns. Locally advanced rectal tumors, due to their location in the rectal wall, often present as irregular, deformable, and subject to significant day-to-day (interfraction) variations.

Current standard treatment for locally advanced rectal cancer includes neoadjuvant (chemo-) RT followed by surgery. Depending on clinical stage, the RT consists of either short course (25 Gy in 5 fractions) (SCRT) or long course RT (45–50.4 Gy in 25–28 fractions) (LCRT), to the primary clinical target and elective lymph node region (Kapiteijn *et al* 2001, Sebag-Montefiore *et al* 2009, Glynne-Jones *et al* 2017). Dose escalation, as boost to the primary tumor, has been proposed to increase local control, especially in a non-surgical management context (Dossa *et al* 2017, Appelt *et al* 2020, Verheij *et al* 2024).

Delivering an effective RT boost requires understanding the day-to-day variation of the primary tumor to determine accurate and robust planning target volume (PTV) margins. Limited data are available on estimating daily variations of the primary gross tumor volume (GTVp) in rectal cancer patients, with existing studies employing diverse methods. A study by Kleijnen *et al* analyzed daily variations by measuring the variation of center-of-mass (COM) of the tumor (2019). However, relying on COM as a surrogate for tumor motion assumes that the tumor is rigid and does not necessarily take into account rotational transformations and local deformations. Other studies have used surface-based deformable methods (Brierley *et al* 2011, Kleijnen *et al* 2016). For this purpose, several different metrics exist to evaluate surface distance measures. In particular, the bidirectional local distance (BLD) has been proposed as a robust measure in the setting of complex local deformations (Kim *et al* 2012), and has been used in a prior study to evaluate rectal tumor displacements (Kleijnen *et al* 2018). To our knowledge, no previous studies have evaluated and compared different methods for estimating the positional variations of the primary tumor volume in rectal cancer patients.

This study aims to quantify interfraction shape and positional variations of the primary tumor volume in LCRT treatments by assessing systematic, random, and group mean (GM) errors, applying two quantification strategies: COM variation and surface variation using the BLD measure.

## 2. Methods

### 2.1. Clinical study design

**Figure 1.**
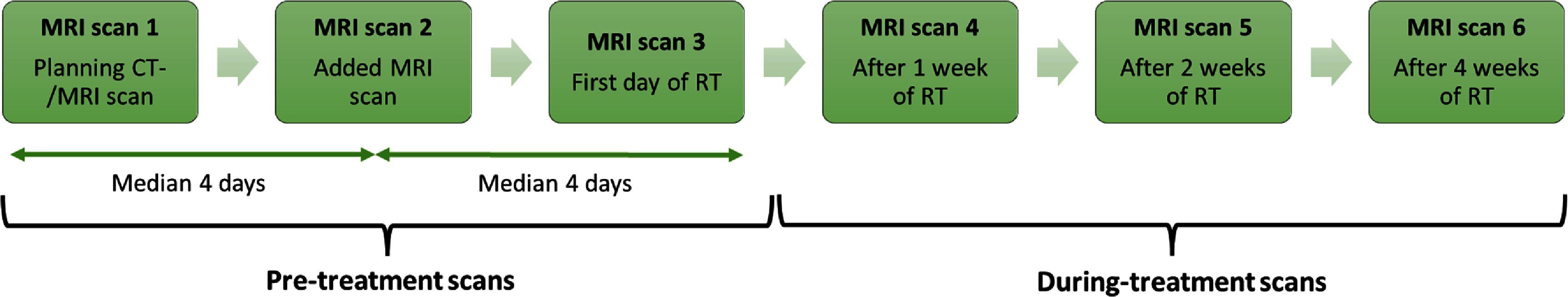
Flowchart illustrating the time-points of the six MRI examinations.

The current work was based on a prospective clinical imaging study (AMPERE) of repeat MRI scans before and during RT for rectal cancer. Inclusion criteria for the study were patients (above 18 years old) referred to standard chemoradiotherapy for locally advanced rectal cancer (50.4 Gy in 28 fractions, concomitant Capecitabine on treatment days). The main exclusion criteria included prior surgery in the minor pelvis region and contraindications based on MRI safety requirements. A comprehensive list of inclusion and exclusion criteria can be found in the appendix (table A.1). Patients underwent a total of six MRI examinations each. The first three MRIs were performed before the start of RT, while the last three were performed during the RT course; specifically, after one, two, and four weeks of RT (figure 1).

The MRI examinations consisted of 3D T2-weighted (1.5 × 1.5 × 1.5 mm^3^) and diffusion-weighted imaging (DWI) (2.5 × 2.5 × 4.0 mm^3^) acquired on a RT dedicated Philips Ingenia 3 T MRI scanner. The MRI scan acquisition parameters are provided in appendix A. 2. Patients were instructed to empty their bladder before scanning in line with the bladder filling protocol used for rectal cancer patients in the department. The patients were scanned in supine position, with standard fixation equipment for all scans. Prior to imaging, hyoscinbutylbromid (antispasmodic to reduce bowel peristalsis) was administered, if not contra-indicated. No bowel preparation or bowel filling evaluation was performed.

The study protocol was approved by the Regional Scientific Ethics Committee for Northern Denmark (N-20170 064). All patients provided written informed consent and the study was registered on ClincalTrials.gov (NCT03619668).

### 2.2. Delineations

#### 2.2.1. Primary GTVp

The primary GTVp was manually delineated on the 3D T2-weighted images for each time point (MRI scan 1-6). The DWI images and calculated apparent diffusion coefficient maps were used to support the evaluation of tumor extent. However, since DWI images are prone to geometrical distortions, the tumor definition primarily relied on the 3D images to obtain the most geometrically accurate representation of the tumor. This was a multidisciplinary process involving a senior consultant oncologist, a senior consultant radiologist, and a medical physicist. The consultant radiologist, who participated in the initial evaluation, also reviewed and approved all final tumor delineations.

#### 2.2.2. Image registration

**Figure 2.**
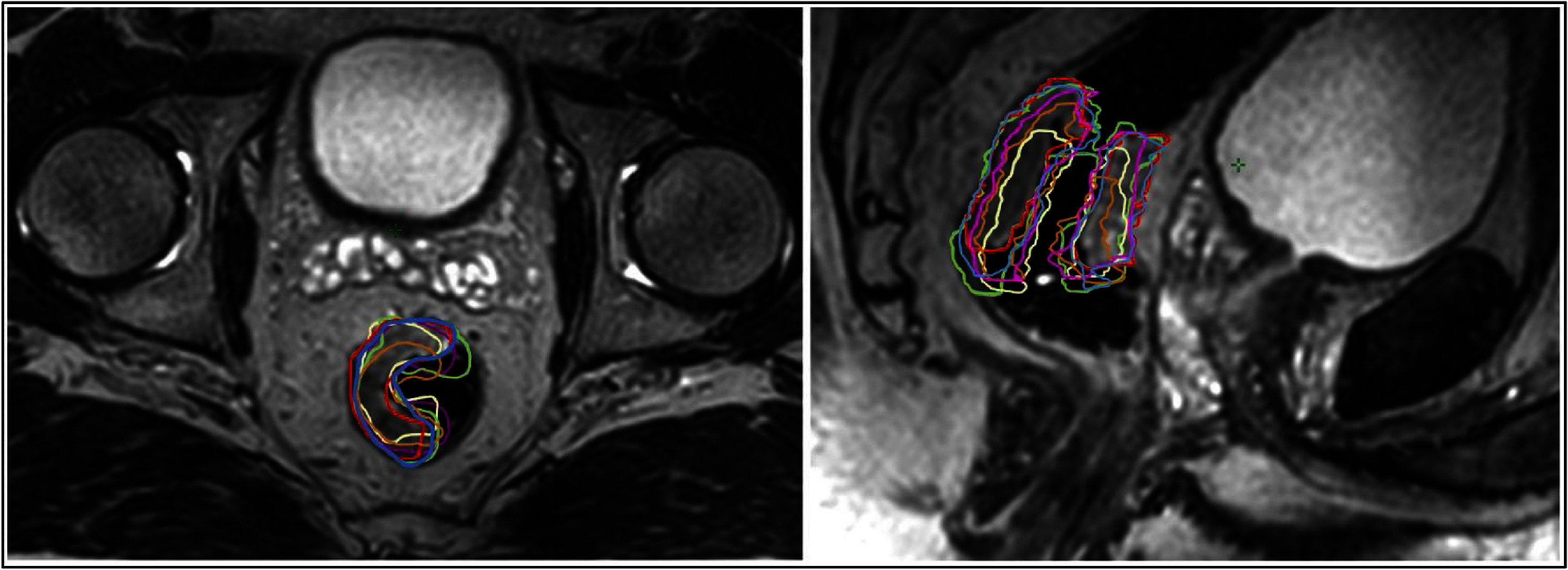
Left: axial view of six GTVp delineations transferred by rigid bone registration to the baseline 3D MRI scan. Right: Same delineations in the sagittal plane. Each color corresponds to a delineation on one of the six MRI scans.

**Figure A.3.**
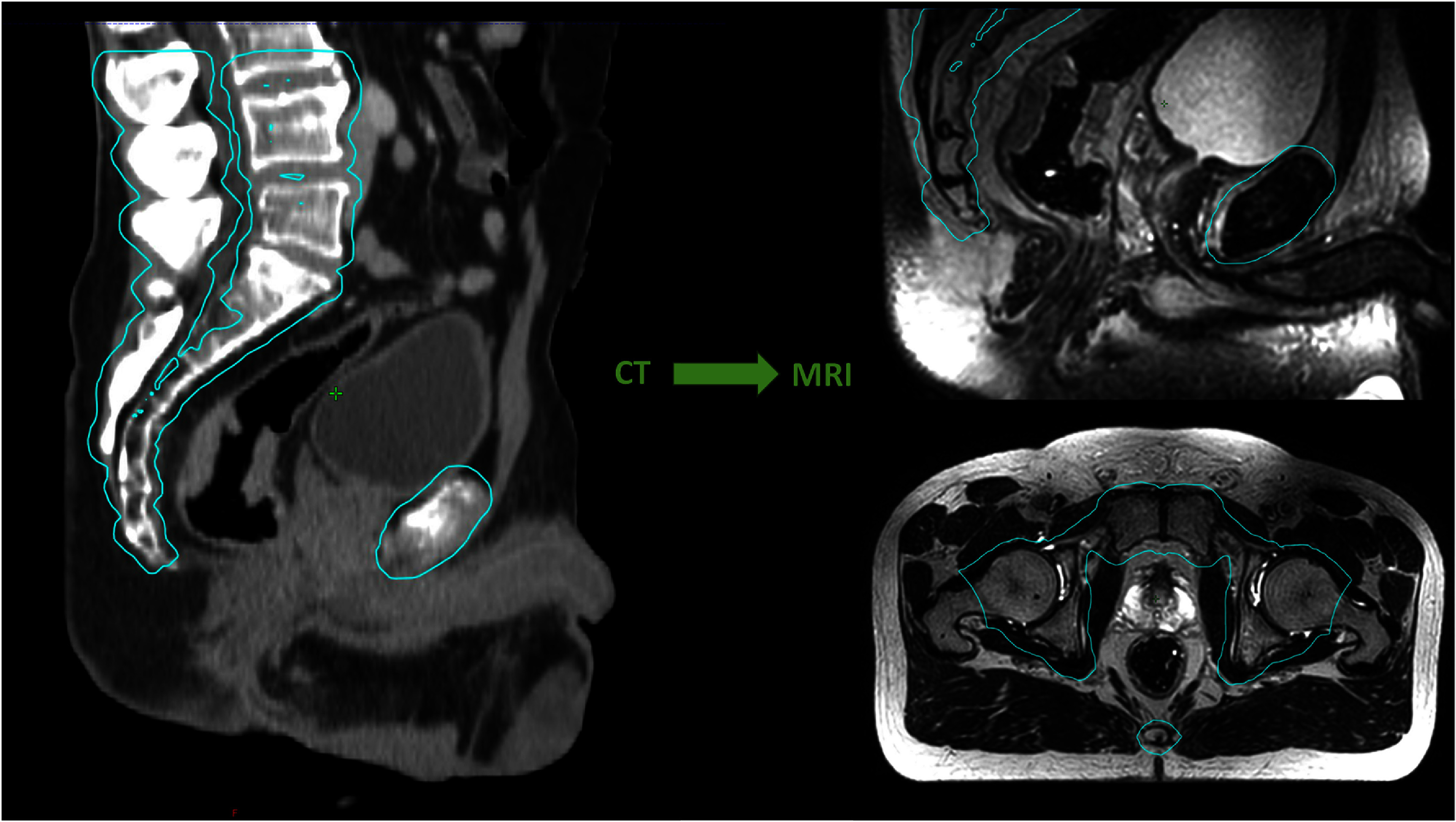
Example of segmentation of pelvic bone structures used to create a mask for MRI scan registration. Left: Sagittal view of CT scan showing auto-segmented bony structures (cyan) with a 5 mm margin. Right: Axial and sagittal views of the mask, transferred to baseline MRI, used for MRI co-registration based on grayscale values within the mask.

The positional variation of the GTVp was calculated relative to the initial baseline scan (MRI scan (1). To accomplish this, MRI scans 2 through 6 were each rigidly co-registered to the baseline scan. This was done by creating a mask around the pelvic bone structures (automatically segmented on the planning CT) and registering the MRI images based on grayscale values within the mask (illustrated in figure A.3 in the appendix). The registrations accounted for both translational and rotational displacements (up to approximately 3 degrees). The rigid registrations were subsequently used to transfer each delineated GTVp to the baseline scan (see figure 2). Finally, any registration-induced artifacts, such as small voids within the structures, were post-processed and removed.

### 2.3. Position variation analysis

Positional variation of the GTVp was determined using two different approaches: COM-based and point-based surface displacements.

#### 2.3.1. COM displacement

The COM was calculated for each GTVp on each scan using the department’s clinical RT treatment planning system ECLIPSE™ (Varian Medical Systems, Palo Alto, CA). The displacement of the COM positions for the structures delineated on each MRI scans were subsequently calculated relative to the baseline scan based on the rigid bone registrations.

#### 2.3.2. Point-based surface displacements

**Figure 3.**
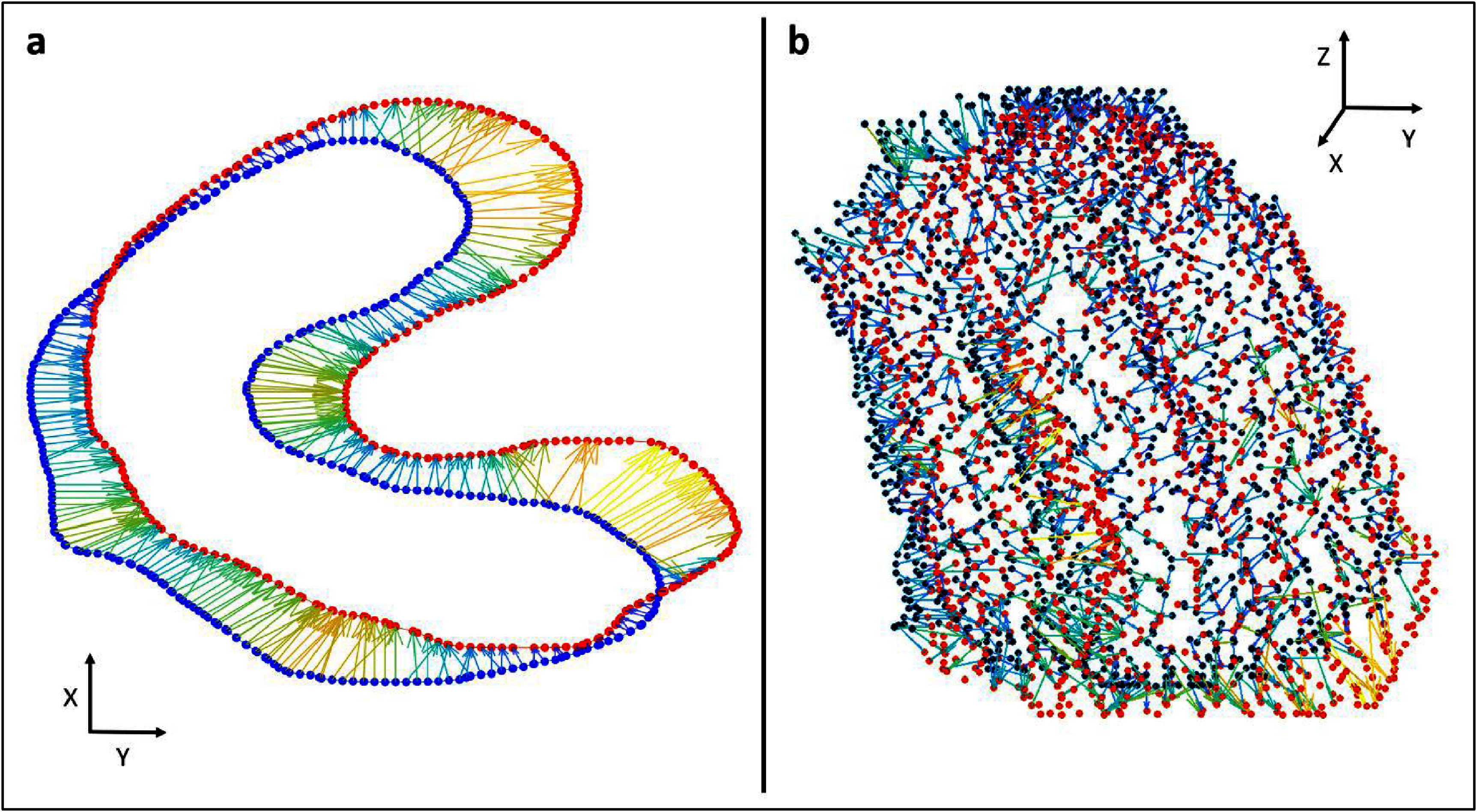
a: Illustration of the BLD measure. Blue dots represent surface points on the baseline GTVp and red dot a GTVp from a subsequent scan. The connecting arrows represents the connections found by the BLD algorithm. This is a simplified version of the algorithm restricted to 2D. b: Down-sampled 3D representation of baseline GTVp and a subsequent GTVp. with the BLD visualized in 3D.

To determine the surface displacements of the GTVp’s, an in-house script was developed in MATLAB® (MATLAB version: 9.14.0 (R2023a), Natick, Massachusetts: The MathWorks Inc.; 2023.). The script calculated point-based surface variation using the BLD measure and was based on an BLD algorithm available in the MathWorks File Exchange repository (Rørtveit 2021). The algorithm follows three main steps, performed for each surface point on the baseline structure (blue points in figure 3) relative to each structure from the subsequent scans (test structure; red points in figure 3). Initially, it identifies the nearest distance from the baseline surface point to a point on the test structure. Subsequently, it identifies every point on the test structure for which the selected baseline point is the nearest. This results in a set of candidate points to represent the BLD measure. Finally, the algorithm selects the point (from the candidates) with the largest distance as the resulting mapped BLD point and distance. For each patient, the baseline structure set, encompassing all co-registered GTVp structures, was evaluated as three-dimensional surface points. For every surface point, the distance between its position on the baseline scan and each subsequent MRI scans was computed as a 3D displacement vector using the BLD measure (figure 3(b)). The vectors were then decomposed into *x-, y* and *z*-components, corresponding to left–right, anterior–posterior (AP) and cranial–caudal (CC) direction, respectively. Conclusively, this results in five vector lengths, calculated, and mapped at each baseline surface-point for each direction. The in-plane components (*x* and *y*), were then signed, based on whether the displacement moved out of (positive) or into (negative) the baseline structure, while the sign of the *z*-component was determined in relation to the COM of the baseline structure.

The intra-patient surface variation was quantified by calculating the median, interquartile range (IQR) and 95th percentile in each direction for all surface-points to represent the total (global) surface displacement.

**Figure 4.**
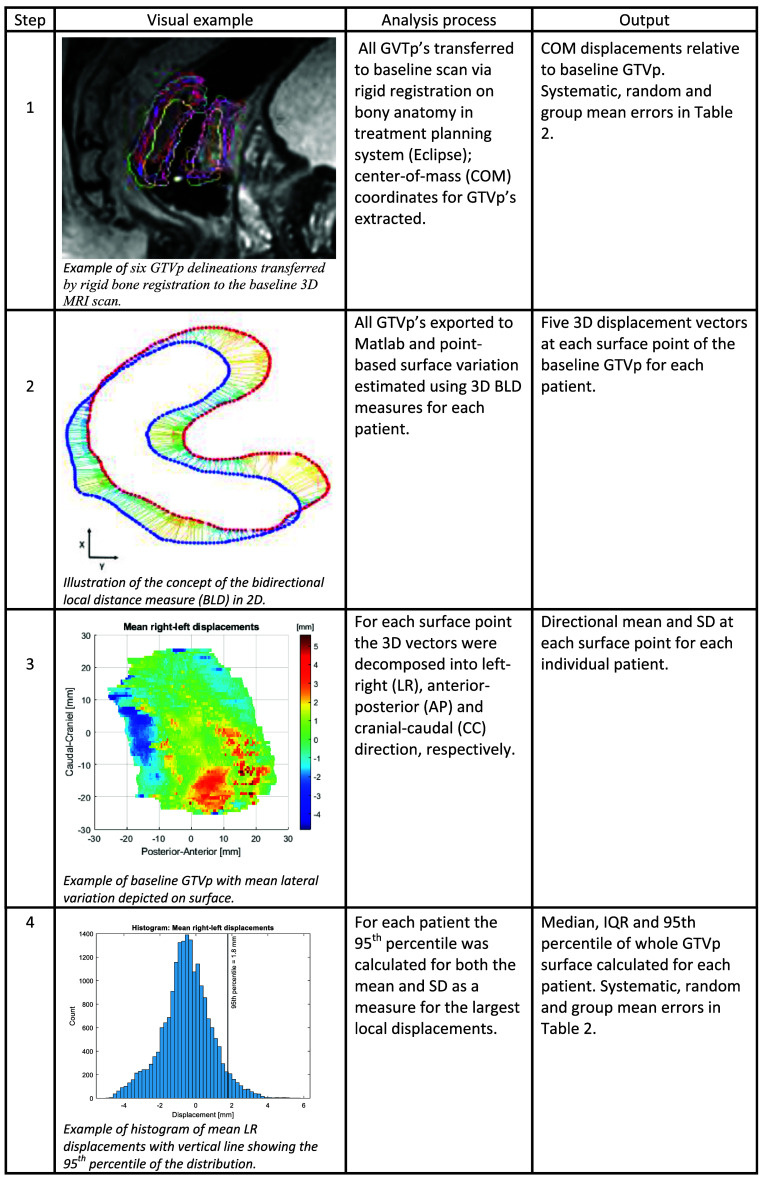
Description of each step involved in the calculation and mapping of systematic, random and group mean errors of the primary gross tumor volume (GTVp).

To evaluate local left, right, anterior, and posterior surface variation relative the tumor’s height in the rectum, a reference rectum structure was generated, featuring 120 equidistant surface points per slice. Baseline GTVp volumes, and their associated set of displacement vectors, were mapped onto the reference geometry based on the relative central axis distance (CAX) from the anal verge. Then each point was mapped to a specific point using the BLD measure. To assess variations in the anterior, posterior, left and right directions, each reference slice was segmented into directional sections, each spanning 180° and comprising 60 points. Additionally, to examine the impact of tumor height, the reference rectum was divided into three sections, each analyzed separately. A flow diagram illustrating the comprehensive analysis process is presented in figure 4. In this analysis, only the top- and bottom slices of the baseline tumor were evaluated in the CC direction.

### 2.4. Systematic, random and GM errors

Systematic, random and GM errors were calculated for the patient population, using the methodology introduced by Van Herk (2004). These calculations were performed for both the COM measurements and the surface displacements. Initially, the analysis was conducted based on the 95th percentile mean and standard deviation (SD) across the entire volume for each patient. Subsequently, the calculations focused on the 95th percentile for segments, which were defined relative to the location of surface points on a reference rectum structure. This allowed for an analysis of both overall and localized displacement errors.

## 3. Results

The study included a total of 16 patients diagnosed with locally advanced rectal cancer (T2-4, N0-2) in the period from 2018 to 2021 at Aalborg University Hospital, Denmark. All patients completed the six planned MRI examinations, resulting in a total of 96 MRIs for analysis. Table 1 shows the general patient characteristics of the patient population. The median interval between the individual pre-treatment MRI scans (MRI scan 1–3) was four days. All patients underwent LCRT, receiving 50.4 Gy in 28 fractions, and concurrent chemotherapy with capecitabine. No consolidation or induction chemotherapy was administered. The treatment plans were delivered as 7 or 9-field IMRT, and daily image guidance RT was conducted using stereoscopic kV-imaging with bone alignment and six degrees of freedom positioning, allowing up to 3 degrees rotational compensation.

**Table 1.** Patient and primary gross tumor volume (GTVp) characteristics.

Number of patients		16
Age	Median	63
Sex	Female	9
	Male	7
cT category	T2	1
	T3	10
	T4	5
cN category	N0	2
	N1	8
	N2	6
Location of GTVp	Low	13
	Mid	3
GTVp volume at baseline	Median (cm^3^)	28.2
	Interquartile range (cm^3^)	36.4

The systematic, random a GM errors for the COM measurement can be seen in table 2. The systematic error was 1.7, 1.3 and 2.0 mm for AP, right–left (RL), and CC direction, respectively. The random error was 2.1, 1.2 and 2.2 mm, while the GM errors were −0.3, 0.4 and −0.3 mm for AP, RL, and CC directions, respectively.

**Table 2.** Systematic, random and group mean errors for center-of-mass (COM) and surface measurements (based on 95th percentiles). For the COM the sign of the group mean (GM) describes the direction of the shifts where positive values correspond to shifts in the cranial, left and posterior direction. For the surface the GM sign either describes an inward (negative) or outward (positive) deformation.

	Center-of-mass:			Surface (95th percentile):		
	AP	RL	CC	AP	RL	CC
Systematic error (mm)	1.7	1.3	2.0	0.4	0.5	1.0
Random error (mm)	2.1	1.2	2.2	2.8	3.0	2.9
Group mean (mm)	−0.3	0.5	−0.3	1.1	1.1	1.8

**Figure 5.**
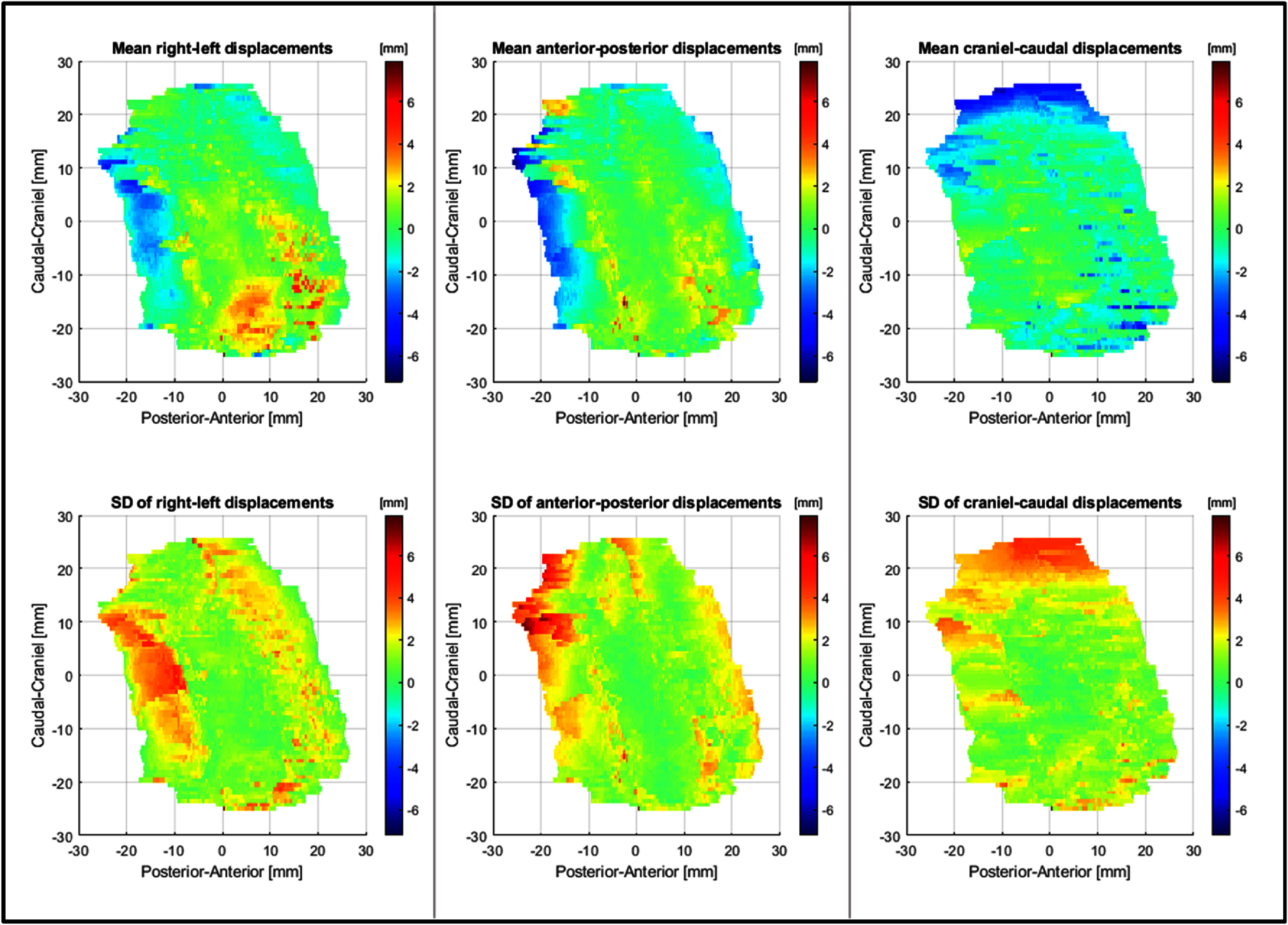
Detailed view of the spatial variability across the surface of the baseline structure for a single patient; shown as mean and standard deviation (SD) decomposed in left/right (column 1), anterior/posterior (column 2), and cranial/caudal (column 3) displacements.

For the analysis of surface displacements, the delineated GTVp’s were each represented by 7000–28 000 surface points, varying according to tumor size. Figure 5 illustrates the mean and SD for the displacements in each direction for a single patient, providing a detailed view of the spatial variability.

Using the 95th percentile mean and SD across the entire volume for each patient the random, systematic, and GM errors were calculated, as shown in table 2. The systematic error was 0.4, 0.5, and 1.0 mm for AP, RL, and CC direction, respectively. The random error was 2.8, 3.0 and 2.9 mm, and the GM errors were 1.1, 1.1 and 1.8 mm for AP, RL, and CC directions, respectively. The mean and SD for each patient can be seen in table A.4, in relation to the COM measurements.

**Figure 6.**
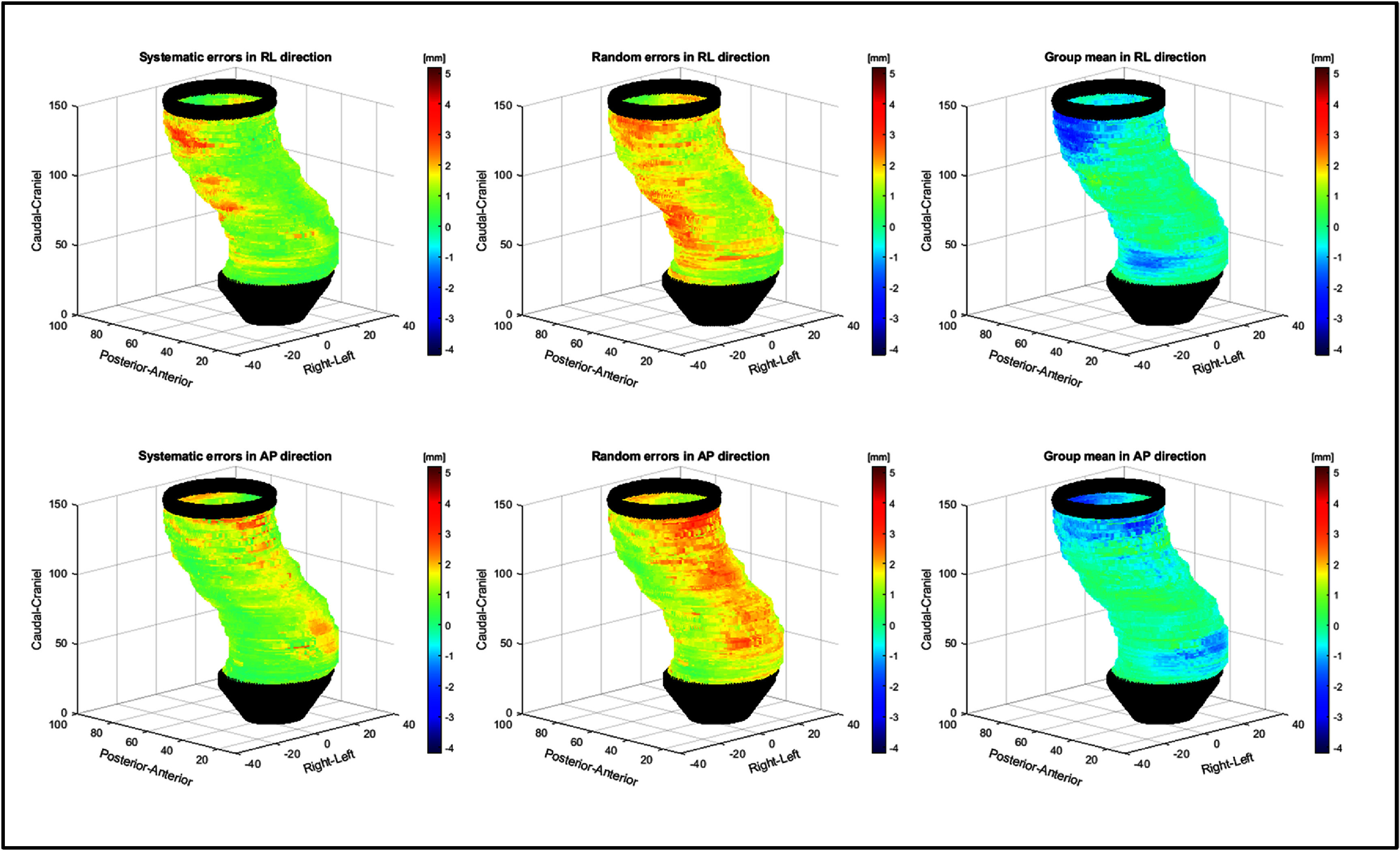
Visualization of the systematic, random and group mean errors in left/right (LR) and anterior/posterior (AP) direction for the entire cohort. Black points represent positions where not enough data-points were present for analysis.

For the surface displacements projected onto a reference rectum, the random, systematic, and GM errors are visually represented in figure 6. Since the tumors were in different locations (heights), each slice on the reference rectum was comprised of a varying number of inter-patient data points, ranging from 1 to 12, depending on the number of overlapping slices with tumor. A minimal threshold of 5 overlapping slices was established when calculating inter-patient variation, resulting in a datapoints from 2.8 to 13.0 cm along the CAX of the reference rectum. By segmenting the rectum into patches characterized by height (low: 28 to 51 mm, mid: 52 to 100 mm, and high: 102 to 130 mm) and direction (anterior, posterior, left, and right), we calculated the median, IQR, and 95th percentile for each segment, detailed in table 3. Here the 95th percentile systematic error ranged from 1.2 to 2.6 mm, depending on direction and location. The 95th percentile random error ranged from 1.8 to 3.7 mm, while the GM errors were between −1.6 and 2.1 mm, depending on direction and location.

**Table 3.** Systematic (∑), random (*σ*) and group mean (GM) errors for anterior, posterior, right, left, cranial and caudal direction. Divided into section relative to the location in the rectum (low: 28–51 mm, mid: 52–100 mm and high: 102–130 mm). IQR: interquartile range.

	Anterior			Posterior			Right			Left			Cranial	Caudal
Direction Section	Low	Mid	High	Low	Mid	High	Low	Mid	High	Low	Mid	High		
Median ∑ (mm)	1.2	1.4	1.4	0.7	1.1	1.2	0.9	1.1	1.3	0.7	1.1	1.1	2.5	2.0
IQR (mm)	0.8	0.7	0.7	0.5	0.7	0.8	0.6	0.6	0.9	0.5	0.7	0.8	0.1	0.2
95th percentile (mm)	2.1	2.0	2.1	1.8	1.8	2.3	1.6	2.0	2.5	1.2	2.2	2.4	2.6	2.2

Median *σ* (mm)	1.7	1.4	1.4	1.3	1.6	1.8	1.4	1.5	1.5	1.2	1.5	1.6	3.4	2.2
IQR (mm)	0.7	0.5	0.6	0.7	0.7	0.7	0.8	0.7	0.8	0.6	0.7	0.6	0.3	0.1
95th percentile (mm)	2.4	2.1	2.3	2.0	2.4	2.9	2.2	2.5	2.4	1.8	2.2	2.3	3.7	2.3

Median GM (mm)	−0.3	−0.6	−1.0	−0.4	−0.2	−0.5	−0.4	−0.2	−0.9	−0.2	−0.5	−0.3	−1.8	1.9
IQR (mm)	0.5	0.5	0.7	0.6	0.3	0.8	0.5	0.4	1.0	0.4	0.6	0.5	0.2	0.2
95th percentile (mm)	0.3	−0.1	0.0	0.1	0.3	0.4	0.1	0.3	0.0	0.4	0.2	0.3	−1.6	2.1

## 4. Discussion

The aim of this study was to quantify interfraction shape and positional variations of primary tumor volumes and to present and compare two methods for quantifying theses positional variation in primary rectal tumors. We examined interfraction position variation of the GTVp relative to bony structures for patients treated with LCRT. This was done based on both the COM and point-based surface variations. In assessing surface variations, we initially applied the 95th percentile for mean and SD across the entire tumor volume of each patient to determine global random, systematic, and GM errors. Our findings indicated that, compared to the COM, the random errors increased, when comparing to the 95th percentile surface displacements. An increase in random and GM errors is expected if local displacements, which might not influence the COM, are present. However, by applying the 95th percentile, we are selecting regions with the largest positive mean and SD for each patient, without considering specific locations. This approach reduces interpatient variation, which may lead to an underestimation of systematic errors and an overestimation of random errors, indicating a limitation of global estimations compared to local assessments. By mapping surface displacements from individual patients to a reference geometry we were able to calculate the 95th percentile of local left, right, anterior, and posterior surface variations relative the tumor’s height in the rectum. In this analysis, the systematic and random errors generally showed an increase for most locations and directions when compared to the results obtained through COM calculations. Furthermore, a general tendency that tumors located high in the rectum experience larger variation is seen when evaluating the 95th percentile, in line with findings in other studies (Chong *et al* 2011, van den Ende *et al* 2019).

A small number of studies have investigated interfraction motion of the GTV for rectal cancer patients in different settings and using a range of methodologies (Brierley *et al* 2011, Kleijnen *et al* 2016, 2019, van den Ende *et al* 2019, Rosa *et al* 2021). An overview of these studies, detailing their study setups and methodologies, is provided in table 4.

**Table 4.** Overview of studies evaluating positional variation of rectal cancer tumors. TMN: Tumor, Node and Metastasis. RT: radiotherapy. LRCT: long course chemo-RT. SCRT: short course RT. CBCT: cone beam computed tomography, MRI: magnetic resonance imaging, COM: center-of-mass.

Reference	No. patients	TNM	RT	Position	Imaging	Imaging schedule	Analysis method
Rosa *et al* (2021)	32	T2-4 N0-2 M0-1	LCRT	16 prone 16 supine	CBCT	1 per day for first 5 fractions, then 1–2 per week.	Not specified (‘*performed on Raystation Platform*’)
van den Ende *et al* (2019)	19	T2-3 N0-2 M0	11 SCRT 8 LCRT	Supine	CBCT + 2xMRI	Median: 1st MRI: start of RT, 2nd MRI: 7 d after. CBCT: daily pre- + post-treat CBCT + for LCRT weekly pre-treat CBCT	Interfraction displacement of COM relative to bony anatomy on MR2 with respect to MR1.
Kleijnen *et al* (2019)	16 + 16	T2-4 N0-2 M0-1	16 SCRT 16 LCRT	Supine	MRI	SCRT: daily and LCRT: weekly	COM measurement with calculated distance vectors from baseline scan.
Kleijnen *et al* (2016)	16	T2-3 N0-1 M0-1	SCRT	Supine	MRI	Daily	Deformation vector field for every delineated surface voxel. Elastix with non-rigid B-spline transformation.
Brierley *et al* (2011)	17	Stage II or III	LCRT	Prone	CT	Week 1, week 3 and week 5.	Finite-element model-based deformable image registration measuring 3D spatial change.

Particularly, the works of Kleijnen *et al* and Brierley *et al* stand out for comparison with our study, as they specifically investigated COM- or surface-based variations using either MRI or therapeutic CT imaging, respectively.

Brierley *et al* assessed the interfraction motion based on spatial change of the GTV surface using finite-element model-based deformable image registration (2011) for 17 rectal cancer patients treated with LCRT. They found a systematic error of 1.9–4.0 mm, 2.1–2.7 mm and 3.5–4.5 mm for AP, RL, and CC direction, respectively. The random error was 3.8–6.3 mm, 2.0–2.1 mm and 5.4–6.3 mm, and GM errors of 0.9, 1.1 and 1.6 mm were reported, in the AP, RL, and CC directions, respectively. However, the study was based on delineation on three CT scans (after 1, 3 and 5 weeks of RT), and the group reported uncertainties in the later scans due to lack of contrast.

Kleijnen *et al* assessed interfraction motion of the primary tumor for both SCRT and LCRT rectal cancer patients (2019). The authors evaluated systematic, random and GM errors for 16 LCRT patients based on the same COM concept as used in this study. They found a systematic error of 3.6, 2.3 and 4.8 mm for AP, RL, and CC direction, respectively. The random error was 2.5, 1.5, and 3.3 mm, while the GM errors were −1.3, 1.1 and −0.4 mm for AP, RL, and CC directions, respectively. These are considerably larger than the findings in the present study. This could be due to the timepoints of the MRI scans, where Kleijnen *et al* used weekly imaging throughout the RT course. It could also be due to differences in the delineation process, which might itself introduce inter-scan variations, including the fact that their GTV delineations were based on 2D T2-weighted scans with a slice thickness of 3 mm (compared to the isotropic 1.5 mm 3D MRI scans in the present study). In an earlier study, the group also investigated tumor surface deformation over different timescales in rectal cancer patients receiving short-course RT (Kleijnen *et al* 2016). In the study, a non-rigid B-spline transformation was used for image registration. However, since this approach relies on grayscale registration, the methodology may be less effective when contrast varies between time points on MRI scans. This limitation is particularly relevant for rectal cancer tumors treated with long-course RT, where treatment-induced changes such as edema and fibrosis influence signal intensity (White *et al* 2021).

To our knowledge, no previous studies have investigated local surface deformation in rectal cancer patients treated with LCRT using high-resolution 3D MRI scans, nor have they compared different methodologies for quantifying the positional variation of primary rectal tumors. Determining whether a COM- or surface-based method provides a more accurate measure of tumor displacement is challenging. In our work, we observed considerably smaller variations for both the COM and surface-based variation analysis compared to findings from other studies. However, we found that the random, systematic and GM errors generally increased when evaluating local 95th percentile surface-variation compared to the COM. Therefore, for tumors that undergo significant local deformations, a surface-based evaluation might be crucial. While our study employed the established BLD measure to estimate surface displacements, accurately capturing the complex movement patterns of irregular tumor volumes presents challenges. Consequently, uncertainties, including surface mapping errors and variations in delineation, become more pronounced when using the 95th percentile as a measure for surface displacements, potentially leading to an overestimation of surface displacement in our analysis. Given these considerations, a COM approach may offer greater robustness, as it is less influenced by local delineation errors.

When including tumor location (low, mid, or high) into our analysis, the systematic errors increased, while the random and GM errors decreased compared to the non-localized surface displacements. This is likely due to local surface displacements becoming less predominant when averaged over the entire patient cohort after being mapped to specific locations. Furthermore, allocating the surface points from each patient to different sections on the reference structure resulted in a limited number of observations/points available for calculating the mean and SD (from 5 to 13).

Ultimately, the main purpose of calculating systematic, random and groups mean errors lies in determining accurate and robust PTV-margins. Accurate PTV margins are essential, as overly large margins may unnecessarily expose organs-at-risk, such as the bladder and small bowel, to high doses, while margins that are too small provide a risk of undertreatment of the primary tumor. This is particularly critical in non-surgical management, where RT is a radical treatment modality. Additionally, understanding individual patient positional variation, including location-dependent behavior, could enable better patient selection for advanced RT techniques such as adaptive RT, including MR-Linac treatments. Our findings suggest that local displacements may be underestimated when using COM-based analyses. Therefore, alternative approaches, such as surface-based analysis, are warranted, particularly for larger tumors where deformation plays a significant role.

Our study was based on data from a prospective clinical imaging study with a balanced gender distribution, using high-resolution MRI scans for precise tumor delineation. However, it was limited by a small patient cohort and the availability of only six MRI examinations per patient for analysis. Additionally, the majority of MRIs were conducted before or early in the RT course (scans 1–4), potentially underestimating treatment effects. The need for a larger patient sample and more comprehensive MRI scan data is acknowledged as a significant limitation that warrants consideration for future research. Furthermore, the study focused on advanced tumors (predominantly T3–T4), which demonstrated considerable local surface variation. These variations might not be present in smaller tumors and more early cancers. Additionally, while the BLD measure has been proposed as a robust method for addressing complex local deformations (Kim *et al* 2012), it has not been specifically validated for locally advanced rectal tumors, and the magnitude of potential errors introduced by the algorithm has not been quantified. Therefore, a validation study should be performed ensure the generalizability of the findings.

## 5. Conclusion

COM-based analysis might underestimate local deformations. Consequently, surface-based methods are recommended for a more robust estimation of the systematic, random and GM errors for PTV-margin calculation. The surface variations tend to increase for tumors located in the upper part of the rectum.

## Data Availability

The data cannot be made publicly available upon publication because they contain sensitive personal information. The data that support the findings of this study are available upon reasonable request from the authors.

## Appendix

**Table A.1.** Inclusion and exclusion criteria for the prospective imaging study AMPERE.

Inclusion criteria:	Patients >18 years referred to standard chemoradiotherapy for locally advanced rectal cancer.
Exclusion criteria:	Prior surgery in pelvic minor region
	Pacemaker
	Neurostimulator
	Other non MR-compatible implants
	Pregnancy
	Incapable of undergoing MRI
	Incapable of understanding the patient information
	Allergic to contrast agent*
	Contraindication for hyoscinbutylbromid
	Reduced renal function (GFR < 50 ml min^−1^)
	*Patients who cannot tolerate the contrast used for DCE-MRI (due to allergies, contraindications for hyoscinbutylbromid or reduced renal function (GFR < 50 ml min^−1^)) will still be offered inclusion in the study, but without the contrast-based MRI scans.

**Table A.2.** Overview of parameters for MRI sequences used in the AMPERE study. Acq: acquisition, Rec: reconstructed, TE: Time to echo, TR: Repetition time, T2W: T2-weighted, TSE: Turbo spin echo, AP: anterior-posterior, RL: Right-left, CC: Cranial–caudal, DWI: Diffusion weighted imaging, EPI: Echo planar imaging.

		Acq. voxel size (mm)			Rec. voxel size (mm)					
Type	Mode	AP	RL	CC	AP	RL	CC	TE (ms)	TR (ms)	Acq. time (m:s)
T2W 2D	TSE	0.75	0.75	3.00	0.48	0.48	3.00	120	5526	3:36
T2W 3D	TSE	1.50	1.50	1.50	0.50	0.50	0.75	197	1500	4:08
DWI	EPI	2.50	2.50	4.00	0.63	0.63	4.00	67	3566	4:35

**Table A.4.** Mean and standard deviation (SD) of the center-of-mass (COM) and surface of the primary tumor in each direction (RL: right–left, AP: anterior–posterior and CC: cranial–caudal) for each patient. For the surface displacements the mean and SD are described by the median, interquartile range (IQR) and 95th percentile (perc.).

				Surface displacements:					
		COM displacements:		Mean (mm)			SD (mm)		
Patient no.	Direction	Mean (mm)	SD (mm)	Median	IQR	95th perc.	Median	IQR	95th perc.
1	RL	−1.4	1.3	−0.6	1.4	1.2	1.3	1.3	3.2
	AP	−2.0	1.7	−0.6	1.7	1.2	1.4	1.2	3.1
	CC	−1.8	2.3	−0.2	1.1	1.1	1.2	1.0	2.8

2	RL	1.4	0.8	−0.5	2.7	2.0	1.7	1.3	3.5
	AP	−2.8	2.8	−0.6	2.3	1.7	1.4	1.1	3.3
	CC	−4.0	3.6	0.5	1.8	3.6	1.6	1.7	4.3

3	RL	0.7	0.5	0.0	0.7	0.9	0.9	0.6	1.8
	AP	0.4	1.9	−0.1	0.7	0.9	1.0	1.0	2.4
	CC	2.0	1.2	0.2	0.6	1.5	0.8	0.7	1.9

4	RL	−0.5	1.0	0.0	0.7	1.2	0.6	0.6	1.7
	AP	1.0	0.7	0.0	0.9	1.3	0.8	0.7	1.7
	CC	−0.3	0.6	0.0	0.6	1.1	0.7	0.6	1.6

5	RL	2.3	1.0	−0.5	1.6	1.8	1.0	1.1	3.3
	AP	2.8	1.3	−0.5	1.9	1.6	1.3	1.3	3.5
	CC	−4.3	2.7	−0.5	1.1	0.9	1.2	1.3	3.6

6	RL	1.9	1.1	−0.3	1.2	1.1	1.3	1.0	2.7
	AP	−2.7	2.4	−0.5	1.5	1.2	1.6	1.4	4.1
	CC	−2.6	2.8	−0.3	1.5	1.1	1.6	1.6	4.8

7	RL	3.2	2.6	−1.3	1.8	0.9	1.9	1.5	4.9
	AP	−0.6	4.6	−1.8	2.8	0.3	2.1	1.9	4.7
	CC	1.2	2.6	0.0	2.4	3.6	1.7	1.3	3.3

8	RL	0.0	0.9	−0.7	1.9	0.8	0.9	1.0	2.5
	AP	−3.2	1.6	−0.8	2.4	1.8	1.1	1.0	2.9
	CC	1.1	2.2	0.6	1.8	3.5	1.2	1.0	3.1
9	RL	−0.3	1.7	−0.2	1.3	1.5	1.4	1.3	3.4
	AP	−2.1	3.5	−0.3	1.2	1.1	1.3	1.4	3.7
	CC	0.2	1.3	0.0	1.4	2.4	1.5	1.2	3.4

10	RL	0.2	0.6	0.0	0.6	0.9	0.5	0.5	1.5
	AP	−0.2	1.0	0.0	0.7	1.2	0.5	0.6	1.8
	CC	1.2	0.9	0.2	0.6	1.5	0.5	0.6	2.0

11	RL	−1.0	1.8	−0.4	1.1	0.8	1.0	1.1	2.5
	AP	0.1	0.9	−0.6	1.3	1.0	1.2	1.1	2.9
	CC	2.6	3.4	0.2	1.1	2.3	1.0	0.8	2.5

12	RL	1.2	0.5	−0.5	1.1	0.8	1.0	0.9	2.4
	AP	0.6	1.5	−0.6	1.1	0.4	1.0	0.9	2.6
	CC	−1.0	2.8	0.0	1.1	2.7	1.1	1.1	3.4

13	RL	0.1	0.5	−0.1	0.6	0.5	0.6	0.6	2.0
	AP	0.9	1.4	−0.1	0.5	0.5	0.5	0.6	1.9
	CC	1.4	1.8	0.0	0.5	1.1	0.6	0.6	2.1

14	RL	−1.4	1.0	−0.3	1.1	0.9	1.2	1.1	3.0
	AP	0.2	1.8	−0.4	1.2	0.8	1.3	1.3	3.4
	CC	−1.8	2.1	−0.2	1.2	1.1	1.3	1.0	2.9

15	RL	1.1	0.3	0.0	0.9	1.2	0.7	0.6	1.7
	AP	1.9	0.6	0.0	1.5	2.1	0.9	0.8	2.0
	CC	0.7	1.1	−0.2	0.9	1.1	0.7	0.7	1.8

16	RL	−0.5	0.7	−0.2	0.7	0.8	0.5	0.6	1.5
	AP	0.9	0.8	−0.1	0.7	0.6	0.5	0.5	1.2
	CC	0.0	0.6	0.0	0.3	0.6	0.4	0.6	1.1
